# Distinct Neurobehavioural Effects of Cannabidiol in Transmembrane Domain *Neuregulin 1* Mutant Mice

**DOI:** 10.1371/journal.pone.0034129

**Published:** 2012-04-03

**Authors:** Leonora E. Long, Rose Chesworth, Xu-Feng Huang, Alexander Wong, Adena Spiro, Iain S. McGregor, Jonathon C. Arnold, Tim Karl

**Affiliations:** 1 Schizophrenia Research Institute, Darlinghurst, New South Wales, Australia; 2 Neuroscience Research Australia, Randwick, New South Wales, Australia; 3 University of New South Wales, Sydney, New South Wales, Australia; 4 Centre for Translational Neuroscience, School of Health Sciences, University of Wollongong, Wollongong, New South Wales, Australia; 5 Department of Pharmacology, University of Sydney, Sydney, New South Wales, Australia; 6 Brain and Mind Research Institute, Sydney, New South Wales, Australia; 7 School of Psychology, University of Sydney, Sydney, New South Wales, Australia; Chiba University Center for Forensic Mental Health, Japan

## Abstract

The cannabis constituent cannabidiol (CBD) possesses anxiolytic and antipsychotic properties. We have previously shown that transmembrane domain *neuregulin 1* mutant (*Nrg1* TM HET) mice display altered neurobehavioural responses to the main psychoactive constituent of cannabis, Δ^9^-tetrahydrocannabinol. Here we investigated whether *Nrg1* TM HET mice respond differently to CBD and whether CBD reverses schizophrenia-related phenotypes expressed by these mice. Adult male *Nrg1* TM HET and wild type-like littermates (WT) received vehicle or CBD (1, 50 or 100 mg/kg i.p.) for 21 days. During treatment and 48 h after withdrawal we measured behaviour, whole blood CBD concentrations and autoradiographic receptor binding. *Nrg1* HET mice displayed locomotor hyperactivity, PPI deficits and reduced 5-HT_2A_ receptor binding density in the substantia nigra, but these phenotypes were not reversed by CBD. However, long-term CBD (50 and 100 mg/kg) selectively enhanced social interaction in *Nrg1* TM HET mice. Furthermore, acute CBD (100 mg/kg) selectively increased PPI in *Nrg1* TM HET mice, although tolerance to this effect was manifest upon repeated CBD administration. Long-term CBD (50 mg/kg) also selectively increased GABA_A_ receptor binding in the granular retrosplenial cortex in *Nrg1* TM HET mice and reduced 5-HT_2A_ binding in the substantia nigra in WT mice. *Nrg1* appears necessary for CBD-induced anxiolysis since only WT mice developed decreased anxiety-related behaviour with repeated CBD treatment. Altered pharmacokinetics in mutant mice could not explain our findings since no genotype differences existed in CBD blood concentrations. Here we demonstrate that *Nrg1* modulates acute and long-term neurobehavioural effects of CBD, which does not reverse the schizophrenia-relevant phenotypes.

## Introduction

Cannabis abuse is linked with a moderate increase in the risk of developing schizophrenia [1] although this relationship has been discussed controversially in the field [2]–[5]. The association of a catechol-O-methyltransferase gene polymorphism with increased psychotic symptom occurrence after adolescent cannabis use [6] suggests that the extent and nature of the schizophrenia-relevant behavioural effects of cannabis may have a genetic underpinning. Δ^9^-tetrahydrocannabinol (THC) is the most abundant of the >70 cannabis constituents and is responsible for the euphoric and psychotomimetic effects of cannabis. Cannabidiol (CBD) is another major cannabis constituent present in lower abundance than THC in most cannabis samples [7] that is not psychotropic and ameliorates some of the unpleasant psychoactive effects of THC [8], [9]. Therapeutic potential for CBD in treating psychiatric disorders is suggested by reports of its antidepressant [10], [11], anxiolytic- [12], [13] and antipsychotic-like effects [12]–[16] in rodent models. CBD also produces anxiolytic effects in healthy volunteers and those suffering from social anxiety disorders [17]–[19] and some antipsychotic-like effects in schizophrenia patients [20]. While the actions of CBD are not fully understood, it has a multitude of pharmacological effects such as antagonising the effects of cannabinoid receptor agonists [21], [22], behaving as an inverse agonist at cannabinoid CB_2_ receptors [22], blocking the orphan receptor GPR55 [23], inhibiting fatty acid amide hydrolase, and activating transient receptor potential vanilloid type 1 channels [24].

We have investigated the effects of cannabis constituents in the transmembrane domain neuregulin 1 heterozygous mutant (*Nrg1* TM HET) mouse, a model for a schizophrenia susceptibility gene that offers partial construct, predictive and face validity for schizophrenia. These mice show age-dependent locomotor and exploratory hyperactivity [25] [reversible with clozapine [26]], impaired preference for social novelty [27] and cognitive deficits (e.g. contextual fear conditioning [28]). Furthermore, *Nrg1* TM HET mice show altered susceptibility to the neurobehavioural effects of THC [29]–[32]. Here, we aimed to assess the effect of CBD on behaviour and receptor binding profiles in these mice. We hypothesised that CBD treatment would attenuate the hyperlocomotor activity of *Nrg1* mutant mice, which is relevant to the psychomotor agitation observed in the ‘positive’ signs of schizophrenia [33]. Furthermore, we hypothesised that mutant and wild type-like (WT) controls would show differential sensitivity to CBD in a battery of tests relevant to schizophrenia [33] and that these behavioural effects would be accompanied by changes in receptor binding density of neurotransmitter systems known to be involved in these behavioural domains. Our study demonstrates that *Nrg1* modulates acute and long-term neurobehavioural effects of CBD, which does not reverse the schizophrenia-relevant phenotypes.

## Results

At the start of the study *Nrg1* TM HET mice weighed significantly less than their WT littermates [*Nrg1* TM HET = 27.4±0.3 g versus WT = 28.5±0.2 g; t(1,125) = 3.1, *P*<0.01] and this difference continued throughout the testing period. Importantly, there was no effect of CBD treatment on body weight development (data not shown). Also, there were no overt signs of CBD treatment, such as on general home cage activity levels, responsiveness to touch or piloerection.

### Behavioural effects of acute CBD exposure

#### Locomotion and exploration

**Figure 1:**
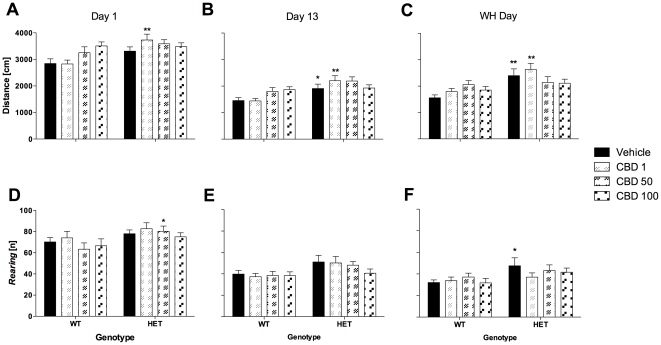
Horizontal locomotor and vertical activity (i.e. *rearing*) in the open field test (10 min) after injection of CBD. A–C: Overall distance travelled and D–F: *Rearing* on days 1, 13 and treatment withheld (WH) day. Data represent mean+S.E.M. Significant one-way ANOVA (split by ‘treatment’) results are shown: * *P*<0.05, ** *P*<0.01 (vs. WT receiving corresponding treatment).

The well-established hyperlocomotor phenotype of *Nrg1* mutant mice was evident on the first test day as measured in the OF [day 1: F(1,117) = 11.3, *P* = 0.001; Fig. 1A]. Further analyses for the different treatment groups revealed that this increase in motor activity of mutant mice was only significant in animals treated with 1 mg/kg CBD, not in those treated with 50 or 100 mg/kg CBD (Fig. 1A). However, there was no significant genotype by treatment interaction. *Nrg1* TM HET mice were also more explorative (i.e. vertical activity) than their WT littermates in the OF [day 1: F(1,114) = 7.6, *P*<0.01; three mice excluded due to equipment malfunction; Fig. 1B]. More specifically, OF exploration of mutant mice was significantly increased in animals treated with an acute dose of 50 mg/kg CBD (Fig. 1D).

#### Anxiety

**Figure 2:**
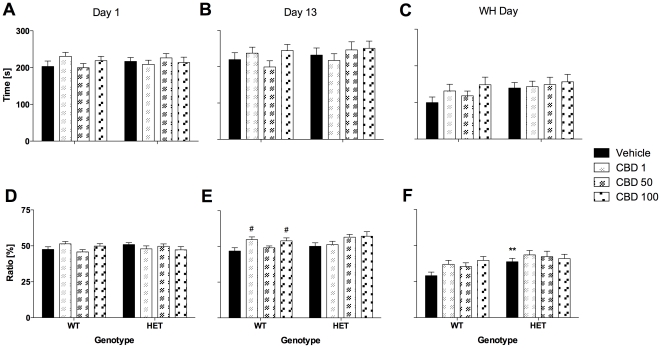
Anxiety-related measures in the open field test (10 min) after injection of CBD. A–C: Time spent in the central area and D–F: Distance ratio on days 1, 13 and treatment withheld (WH) day. Data represent mean+S.E.M. Significant one-way ANOVA (split by corresponding factor) results are shown: # *P*<0.05 (vs. vehicle of corresponding genotype). ** P<0.01 (vs. WT receiving corresponding treatment).

**Figure 3:**
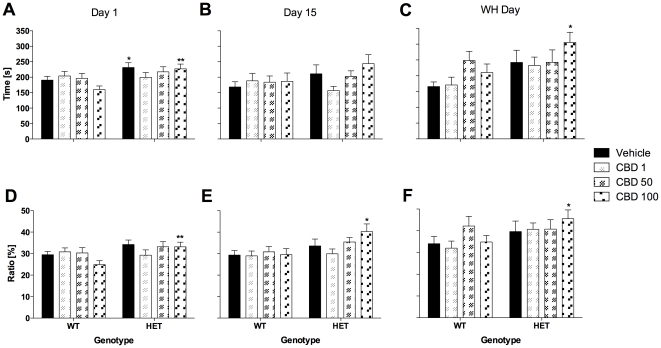
Anxiety-related measures in the light-dark test (10 min) after injection of CBD. A–C: Time spent in the light compartment and D–F: Distance ratio on days 1, 15 and treatment withheld (WH) day. Data represent means+S.E.M. Significant one-way ANOVA (split by ‘treatment’) results are shown: * *P*<0.05, ** P<0.01 (vs. WT receiving corresponding treatment).

Anxiety parameters investigated in the OF (i.e. time spent in the centre and distance ratio) on day 1 were similar for both genotypes and were not affected by CBD treatment (Fig. 2A+D). However, as published previously, *Nrg1* TM HET mice displayed an anxiolytic-like phenotype in the LD test (Fig. 3A+D). Time spent in the light compartment of the LD test was elevated in mutant mice on day 1 [F(1,117) = 9.0, *P*<0.01] compared with control mice. This anxiolytic-like phenotype was statistically confirmed for vehicle-treated mutants and mutants treated with 100 mg/kg CBD (Fig. 3A). Furthermore, *Nrg1* mutant mice displayed an increase in distance ratio in the more aversive light compartment on test day 1 [F(1,115) = 6.6, *P*<0.05; two animals excluded due to equipment malfunction; Fig. 3D]. This genotype difference was only significant in the group of animals being treated with the highest dose of CBD (Fig. 3D). CBD had no impact on anxiety-related measures of the LD test across genotypes.

#### Sensorimotor gating

**Figure 4:**
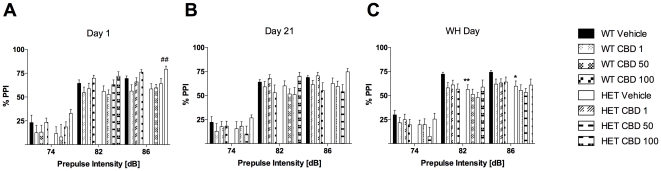
Sensorimotor gating after injection of CBD. A–C: % PPI on days 1, 21 and treatment withheld (WH) day. Data represent means+S.E.M. Significant one-way ANOVA (split by corresponding factor) results are shown: ## *P*<0.01 (vs. vehicle of corresponding genotype), * *P*<0.05, ** *P*<0.01 (vs. WT receiving corresponding treatment).

CBD treatment increased the mean startle response on day 1 [F(3,113) = 11.1, *P*<0.001]. This effect of CBD was evident at a dose of 100 mg/kg in both genotypes (Table 1). As expected, three-way RM ANOVA for ‘prepulse intensity’ confirmed that % PPI increased with increasing prepulse intensity on day 1 [F(2,226) = 432.8, *P*<0.001; Fig. 4A]. Acute CBD had a stimulating effect on % PPI of mice [F(3,113) = 4.7, *P*<0.01]. Specifically, CBD 100 mg/kg increased PPI in *Nrg1* TM HET mice at the 86 dB prepulse intensity compared with vehicle-treated mutant mice (Fig. 4A).

**Table 1:** Startle response.

Day	WT				*Nrg1* TM HET			
	Vehicle	CBD 1	CBD 50	CBD 100	Vehicle	CBD 1	CBD 50	CBD 100
**1**	48.8±4.9	56.0±7.7	51.8±4.9	81.2±6.8 ##	46.8±6.0	37.3±3.2	57.2±10.0	78.8±8.2 ##
**21**	44.4±3.5	50.9±4.9	56.2±5.3	59.7±6.3	50.3±7.0	43.5±4.4	45.4±5.8	59.0±7.9
**WH**	52.8±4.1	54.2±5.3	50.8±5.4	46.3±3.9	47.2±7.3	39.7±3.6 *	34.9±4.7 *	40.4±5.9

Acute CBD (100 mg/kg) increases startle response [arbitrary units] to a 120 dB acoustic stimulus. Data represent means (± S.E.M.). Significant one-way ANOVA (split by corresponding factor) results are shown:

* *P*<0.05 (vs. WT receiving corresponding treatment);

## *P*<0.01 (vs. vehicle of corresponding genotype).

### Behavioural effects of long-term CBD exposure

#### Locomotion and exploration

Hyperlocomotion of *Nrg1* mutant mice was evident on test day 13 [F(1,114) = 18.3, *P*<0.001; Fig. 1B]. This genotype effect was significant in animals treated with vehicle or 1 mg/kg CBD but not in those treated with 50 or 100 mg/kg CBD (Fig. 1B). No interactions were detected. Furthermore, mutant mice exhibited an overall explorative-like phenotype in the OF [F(1,111) = 9.3, *P*<0.01; three mice excluded due to equipment malfunction], although this increase in exploration failed to reach significance in any particular treatment group (Fig. 1E).

#### Anxiety

CBD developed an anxiolytic-like effect in WT mice by day 13 [F(3,114) = 3.6, *P*<0.05], as indicated by an increase in OF distance ratio for the 1 mg/kg and 100 mg/kg treatment groups (Fig. 2E). No genotype-dependent effects were observed in the OF on this test day. *Nrg1* mutant mice displayed an increase in distance ratio in the more aversive light compartment of the LD test on test day 15 [F(1,103) = 7.5, *P*<0.05; eleven mice excluded due to equipment malfunction] but only in those mutant mice that had been treated with 100 mg/kg CBD (Fig. 3E). CBD had no impact on anxiety-related measures of the LD test across genotypes.

#### Social interaction

Social interaction data are presented in Table 2. Overall, CBD treatment increased social interaction as measured by total duration of active social interaction [F(3,112) = 4.7, *P*<0.01]. Furthermore, CBD had a stimulating effect on particular socio-positive behaviours in animals: *nosing* [duration: F(3,112) = 3.6, *P*<0.05] and *anogenital sniffing* [frequency: F(3,112) = 3.7, *P*<0.05 - duration: F(3,112) = 6.2, *P* = 0.001]. Treatment with 50 mg/kg CBD selectively increased total active social interaction time, *nosing* (duration and frequency) and *anogenital sniffing* frequency in *Nrg1* TM HETs. Importantly, CBD at a dose of 50 mg/kg had no concomitant effect on locomotor activity (data not shown). Furthermore, 100 mg/kg CBD increased the duration of *anogenital sniffing* in mutant mice. Finally, *Nrg1* TM HET mice displayed increased frequencies of *nosing* [F(1,112) = 7.7, *P*<0.01]. This genotype effect was significant in mutant animals treated with 1 mg/kg and 50 mg/kg CBD.

**Table 2:** Social interaction.

Parameter	WT				*Nrg1* TM HET			
	Vehicle	CBD 1	CBD 50	CBD 100	Vehicle	CBD 1	CBD 50	CBD 100
*Nosing* [n]	40.7±2.4	41.5±2.4	42.4±3.2	46.1±2.5	43.4±2.9	50.2±3.7 *	52.0±3.5 *	48.2±3.2
*Nosing* duration [s]	46.6±2.0	44.9±4.1	47.8±3.2	55.2±5.1	41.5±2.7	45.3±3.1	62.4±6.0 ##	51.6±5.8
*Anogenital sniffing* [n]	21.1±1.7	18.5±2.2	21.5±2.3	25.2±2.8	17.8±2.0	21.3±2.8	29.4±2.6 ##	26.2±3.4
*Anogenital sniffing* duration [s]	20.3±2.1	19.0±2.8	23.4±3.3	27.2±3.1	14.4±1.9	19.3±3.1	29.9±3.6 ##	23.3±1.9 ##
Total social interaction duration [s]	74.4±5.5	67.1±6.4	76.7±6.8	86.6±7.6	60.2±4.1	67.0±6.1	98.8±10.3 ##	84.8±10.3

Frequency and duration of *nosing* and *anogenital sniffing* with a standard opponent A/JArc mouse after injection with CBD (1, 50 or 100 mg/kg). Data represent means (± S.E.M.). Significant one-way ANOVA (split by corresponding factor) results are shown:

* *P*<0.05 (vs. WT receiving corresponding treatment);

## *P*<0.01 (vs. vehicle of corresponding genotype).

#### Sensorimotor gating

Our analysis did not reveal any significant effects of treatment or genotype on sensorimotor gating. As expected, % PPI increased with increasing prepulse intensity [day 21: F(2,220) = 532.9, *P*<0.001; Fig. 4B].

### Behavioural effects of withholding CBD for 48 h

None of the test mice showed any drug withdrawal-like symptoms (e.g. wet dog shakes [34]) during OF, LD or PPI testing 48 h post final CBD treatment.

#### Locomotion and exploration

The hyperlocomotor phenotype of *Nrg1* mutants was still detectable in the OF on the last experimental day [WH day: F (1,112) = 16.7, *P*<0.001]. This increase in motor activity was significant for mutant mice of the vehicle or 1 mg/kg CBD treatment groups (Fig. 1C). No significant genotype by treatment interaction was found. Despite an overall effect of genotype on exploration [WH day: F(1,105) = 7.6, *P*<0.01; seven mice excluded due to equipment malfunction], only vehicle-treated *Nrg1* HET mice showed a significantly increased frequency of vertical activity compared to WT mice in the OF (Fig. 1F).

#### Anxiety

*Nrg1* TM HET mice were less anxious (i.e. increase in distance ratio in the OF) compared with their WT littermates on WH day [F(1,112) = 8.9, *P*<0.01]. This phenotype was only significantly different between vehicle-treated animals of both genotypes (Fig. 2F). This finding was confirmed in the LD test (Fig. 3). Time spent in the light compartment of the LD test was increased in *Nrg1* HET mice on WH day [F(1,112) = 7.5, *P*<0.01) compared with control mice. This anxiolytic-like phenotype was statistically confirmed for mutants treated with 100 mg/kg CBD (Fig. 3C). In addition, *Nrg1* mutant mice exhibited higher levels of distance ratio in the light chamber of the LD test on WH day [F(1,109) = 4.8, *P*<0.05; three mice excluded due to equipment malfunction]. This genotype difference was only significant in the group of animals being treated with the highest dose of CBD (Fig. 3F). CBD had no impact on anxiety-related measures of the LD test across genotypes.

#### Sensorimotor gating

We detected significant differences between the ASR of WT and *Nrg1* mutant mice on WH day [F(1,111) = 8.3, *P*<0.01]. The startle response was reduced in *Nrg1* TM HET mice treated with 1 mg/kg or 50 mg/kg CBD compared with the corresponding WT groups (Table 1). As on the other test days, % PPI was dependent on the prepulse intensity [WH day: F(2,222) = 498.6, *P*<0.001; Fig. 4C]. Importantly, our analyses detected a PPI deficit in mutant mice on WH day [F(1,111) = 4.8, *P*<0.05], as % PPI was reduced in vehicle-treated *Nrg1* HET mice compared with WT controls at prepulse intensities of 82 dB and 86 dB (Fig. 4C).

### CBD concentration in whole blood

**Figure 5:**
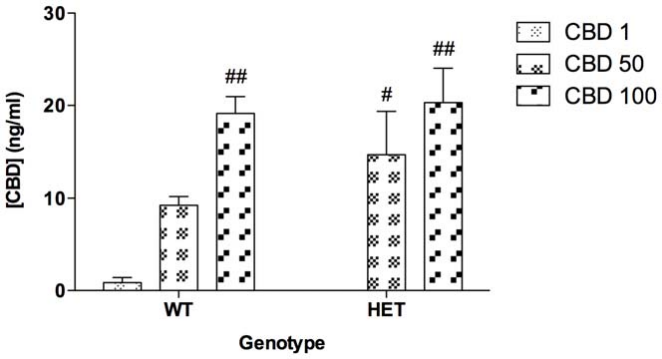
Concentration (ng/ml) of CBD in whole blood 2 days after the last treatment. Data represent means+S.E.M.

GC-MS analysis of CBD in whole blood obtained from *Nrg1* TM HET and WT mice immediately after the final behavioural test, performed 48 h after treatment cessation (48 h after the final of 21 CBD injections: WH day), is depicted in Figure 5. There was no difference between mutant and WT mice in CBD blood concentration, which increased in a dose-dependent manner in both genotypes [F(2,26) = 11.5, *P*<0.001].

### Effects of CBD on autoradiographic receptor binding

**Figure 6:**
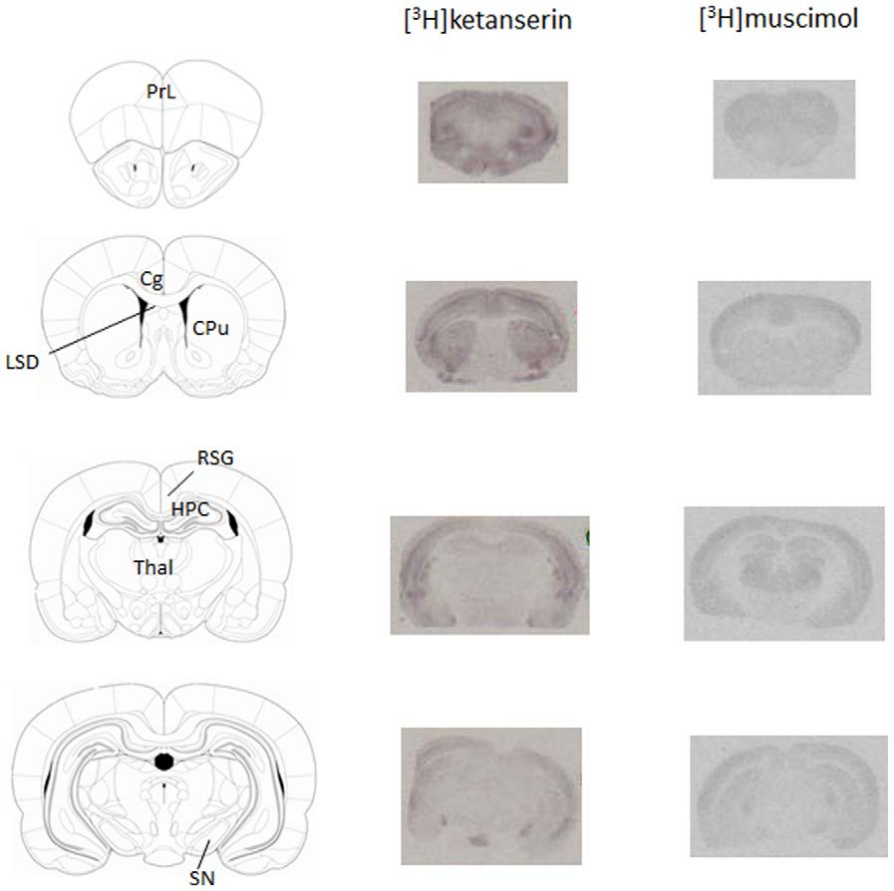
Representative autoradiograms showing [^3^H]ketanserin (5-HT_2A_ receptors) and [^3^H]muscimol (GABA_A_ receptors) binding in specific brain regions. Abbreviations: Cg: anterior cingulate cortex; CPu: caudate putamen; HPC: hippocampus; LSD: dorsolateral septum; PrL: prelimbic cortex; RSG: granular retrosplenial cortex; SN: substantia nigra; Thal: thalamus.

Representative autoradiograms for [^3^H]ketanserin and [^3^H]muscimol binding in WT and *Nrg1* TM HET mice are depicted in Figure 6, and receptor binding data for all radioligands 48 h after treatment cessation are reported in Table 3. There were no changes in CB_1_, 5-HT_1A_ or NMDAR radioligand binding in any brain region (Table 3).

**Table 3:** Specific [^3^H]ketanserin, [^3^H]muscimol, [^3^H]CP 55,940, [^3^H]WAY 100.635 and [^3^H]MK-801 binding in different brain regions.

Region	WT				*Nrg1* TM HET			
	Vehicle	CBD 1	CBD 50	CBD 100	Vehicle	CBD 1	CBD 50	CBD 100
*[^3^H]ketanserin* (5-HT_2A_ receptors)								
Prelimbic cortex	30.4±2.4	30.9±0.3	30.3±1.9	31.3±1.4	31.4±0.9	30.1±0.8	31.3±1.1	31.0±1.0
Cingulate cortex	30.6±0.9	30.6±0.7	30.5±1.2	30.2±0.8	30.7±1.0	31.4±0.5	30.0±0.5	30.1±0.3
Caudate putamen	28.7±0.8	28.4±1.4	29.0±2.5	27.9±0.8	28.1±1.0	29.3±1.0	27.8±0.8	27.8±0.8
Hippocampus	25.8±0.5	25.4±0.5	25.5±0.8	25.3±0.7	25.3±1.1	26.1±0.3	25.2±1.0	25.0±0.9
Substantia nigra	34.6±0.9	33.4±1.8	31.3±1.0#	32.0±2.6	32.3±1.1*	34.1±0.9	33.0±2.0	32.4±0.7
*[^3^H]muscimol* (GABA_A_ receptors)								
Prelimbic cortex	21.8±0.1	21.7±0.8	21.9±1.3	21.5±1.5	20.8±0.9	21.3±1.1	22.2±1.1	21.1±1.9
Cingulate cortex	22.7±0.3	23.0±1.3	23.3±1.4	22.4±0.9	22.3±1.0	22.7±0.6	23.3±1.3	22.3±2.1
Caudate putamen	20.1±0.3	20.2±0.7	20.2±0.8	19.8±0.6	19.7±0.6	20.0±0.4	20.4±0.6	19.7±1.2
Dorsolateral septum	19.7±0.8	19.5±0.7	19.4±0.9	19.7±1.0	19.3±0.5	19.0±0.8	19.2±0.7	19.4±1.2
Retrosplenial granular cortex	20.5±0.8	20.5±0.6	20.2±0.7	20.5±0.8	19.9±0.3	20.4±0.7	21.3±0.6##	20.3±0.5
CA1	22.3±0.9	22.2±1.2	21.6±1.5	22.0±1.2	21.6±1.0	21.6±1.0	22.4±1.2	21.9±1.2
Dentate gyrus	22.3±0.9	22.4±1.1	22.0±1.5	22.5±1.3	22.1±1.5	22.1±0.7	23.0±1.4	22.6±1.3
Thalamus	23.4±1.1	23.4±0.9	23.4±1.6	23.2±1.1	22.7±1.1	22.8±0.7	24.1±1.5	23.0±1.1
*[^3^H]CP 55,940* (CB_1_ receptors)								
Cingulate cortex	27.0±0.6	26.4±0.5	26.8±1.0	26.6±0.6	26.4±0.4	26.8±0.4	27.2±1.0	27.0±0.7
Caudate putamen	26.3±0.3	26.4±0.7	26.6±0.6	26.1±0.8	26.3±0.4	26.8±0.4	26.6±0.3	26.6±0.3
Dorsolateral septum	25.5±0.9	25.3±1.1	25.3±1.1	25.1±1.5	25.4±0.5	25.4±1.2	25.7±0.9	25.8±0.6
Ventrolateral septum	25.4±1.1	25.4±1.4	26.6±1.3	25.7±1.1	26.4±1.0	25.5±1.5	25.8±2.1	25.2±1.1
Hippocampus	26.3±0.5	26.0±0.6	26.1±0.5	25.9±1.2	26.2±0.9	26.2±0.6	26.7±1.0	26.6±0.7
Substantia nigra	105.6±9.7	101.0±5.2	96.4±8.9	97.9±9.6	106.0±5.7	102.5±9.2	102.6±3.2	107.6±12.7
*[^3^H]WAY 100,635* (5-HT_1A_ receptors)								
Cingulate cortex	25.9±1.8	25.5±1.0	26.1±0.7	26.3±1.0	26.1±0.8	25.8±0.9	25.6±1.2	25.5±0.7
Hippocampus	85.3±9.7	86.5±5.7	85.2±6.7	84.5±10.6	86.7±2.8	88.4±5.2	86.3±4.7	83.1±4.6
Lateral septum	31.3±2.1	32.1±1.7	32.3±0.8	31.3±2.3	32.3±1.4	31.9±1.1	31.4±1.4	31.6±0.6
Retrosplenial granular cortex	30.1±3.1	28.6±1.5	28.8±1.5	29.8±2.4	31.1±2.4	30.0±0.7	30.5±1.8	29.3±2.0
Nucleus of the vertical limb of the diagonal band	32.3±3.0	33.2±2.0	33.3±1.1	31.4±2.7	32.0±2.6	34.1±1.3	33.6±1.8	32.8±2.3
*[^3^H]MK-801* (NMDA receptors)								
Cingulate cortex	36.9±0.9	37.8±1.6	38.1±1.7	37.6±0.7	37.5±0.5	38.6±0.7	38.3±1.0	38.1±0.8
Caudate putamen	31.4±1.0	32.1±1.1	32.1±1.3	32.4±0.8	32.2±0.4	32.2±0.5	32.5±1.0	31.8±1.2
Hippocampus	109.9±2.3	111.6±4.6	111.9±5.1	111.2±2.5	110.4±2.3	113.0±3.9	114.8±4.9	111.7±3.6
Dorsolateral septum	34.0±2.2	33.9±1.3	35.5±2.0	34.0±2.1	35.5±0.4	36.0±1.1	33.6±2.5	33.2±1.1
Retrosplenial granular cortex	32.4±1.4	33.2±1.5	33.6±1.6	32.6±1.0	32.8±0.4	33.2±1.0	34.5±1.1	33.5±1.0

[^3^H]ketanserin, [^3^H]muscimol, [^3^H]CP 55,940, [^3^H]WAY 100,635 and [^3^H]MK-801 binding 48 h after the last of 21 daily injections of CBD (1, 50 or 100 mg/kg) (on WH day). Data represent mean binding density nCi/mg tissue (± S.E.M.). Significant one-way ANOVA (split by corresponding factor) results are shown:

* *P*<0.05 (vs. WT receiving corresponding treatment),

# *P*<0.05,

## *P*<0.01 (vs. vehicle of corresponding genotype).

5-HT_2A_ binding in the substantia nigra was reduced in *Nrg1* TM HET mice compared with WT and this effect was dose-dependent, as confirmed by an interaction of ‘genotype’ with ‘treatment’ [F(3,30) = 2.9, P<0.05]. Vehicle-treated *Nrg1* mutants had lower levels of 5-HT_2A_ binding than WT animals. Furthermore, 50 mg/kg CBD decreased specifically 5-HT_2A_ binding in WT mice with no such effect observed in mutant mice (Table 3). There were no changes in 5-HT_2A_ receptor binding in any other region.

GABA_A_ receptor binding was similar across genotypes. However, a significant interaction of ‘genotype’ with ‘treatment’ for the granular retrosplenial cortex indicated, that treatment with 50 mg/kg CBD selectively increased GABA_A_ receptor binding in *Nrg1* TM HET mice compared with vehicle controls [interaction: F(3,31) = 3.0, *P*<0.05]. There were no CBD-induced changes in GABA_A_ receptor binding in any other region (Table 3).

## Discussion

Here we report a range of behavioural effects of acute and chronic CBD in wild type-like and *Nrg1* TM HET mice. CBD had no effect on locomotor activity, although the typical hyperlocomotive phenotype of *Nrg1* TM HET mutant mice was not present after long-term treatment and withholding of CBD (50 and 100 mg/kg). CBD selectively exerted anxiolytic-like effects in WT mice in the OF at both low (1 mg/kg) and high (100 mg/kg) doses. In contrast, high doses of CBD (50 and 100 mg/kg) selectively increased social interaction in *Nrg1* TM HET mice. Acute administration of high-dose CBD enhanced PPI, but tolerance to this effect occurred such that PPI was no longer altered following chronic CBD. *Nrg1* TM HET mice showed decreased 5-HT_2A_ binding in the substantia nigra. CBD did not reverse this change, but enhanced 5-HT_2A_ binding in the substantia nigra in WT mice and increased GABA_A_ density in *Nrg1* TM HET mice.

### Behavioural effects of *Nrg1* genotype and CBD treatment

#### Locomotion and exploration

The lack of effects of acute or long-term CBD on locomotor or exploratory activity is consistent with its negligible motor effects reported for C57BL/6JArc mice, the same inbred mouse strain used as the background for our *Nrg1* model [14]. Baseline locomotor hyperactivity and moderately increased exploratory activity in the OF were evident in *Nrg1* TM HET mice on all three test days, as observed previously [25], [29], [31]. This hyperactivity was retained in *Nrg1* TM HET mice treated with the lower dose of CBD (1 mg/kg), but *Nrg1* TM HET mice treated with 50 and 100 mg/kg CBD did not express either locomotor hyperactivity nor increased *rearing*. Notably, this absence of hyperactivity persisted 48 h after cessation of CBD treatment. Repeated treatment with higher doses of CBD might reduce the potential for the hyperlocomotor phenotype to emerge, which would be in line with reports that acute CBD prevents hyperactivity induced by pharmacological agents such as dexamphetamine [13], [14]. Unfortunately, the response of *Nrg1* TM HET mice to chronic treatment with antipsychotics has not been investigated and the behavioural response of mutant mice to an acute dose of clozapine was task-dependent (i.e. reversal of OF hyperlocomotion but not sensorimotor gating deficits [26]).

#### Anxiety

Vehicle-treated *Nrg1* TM HET mice showed reduced anxiety-like behaviour on test day 1 in the LD test and on the last test day in the OF paradigm. Furthermore, *Nrg1* mutants, who had been exposed to acute or long-term 100 mg/kg CBD exhibited an anxiolytic-like LD phenotype. On the other hand, the anxiolytic effect of long-term CBD (1 and 100 mg/kg) in the OF in WT mice was not present in *Nrg1* TM HET mice, suggesting that the effects of CBD on anxiety-related behaviour are dependent on an intact *Nrg1* transmembrane domain. The fact that anxiolytic-like effects of CBD were only observed in the OF test reflects the importance of the choice of anxiety test used to explore the effects of pharmacological and genetic manipulations, as reported previously in *Nrg1* TM HET [25], [35] and CBD-treated C57BL/6JArc mice [14].

#### Social interaction

There were no pronounced baseline social interaction differences between *Nrg1* TM HET and WT mice, in accordance with our previous observations in adult mice [29], [32]. Interestingly, long-term CBD robustly increased the total active social interaction time and specific social behaviours such as *nosing* and *anogenital sniffing* in *Nrg1* TM HET but not WT mice, at a dose (50 mg/kg) which had no concomitant effect on locomotor activity (data not shown). This selective increase in social behaviour in *Nrg1* TM HET mice suggests that *Nrg1* mutation renders mice more responsive to the facilitatory effects of long-term CBD on social behaviour. Indeed, while CBD has previously been reported to have no outright effect on social interaction in wild type mouse and rat strains [14], [36] it reverses pharmacological deficits in social interaction induced by compounds such as THC [36], [37]. Together, these data suggest that the potential for CBD to improve social function may be unmasked by the *Nrg1* mutation.

#### Sensorimotor gating

Acute CBD (100 mg/kg) selectively increased both PPI and the startle response in *Nrg1* TM HET mice. While it is possible that the enhanced PPI may be due to the concomitant increase in startle reactivity, previous reports have shown that baseline and pharmacologically-induced alterations in PPI are able to be dissociated from startle pulse- or prepulse-elicited reactivity [38], [39]. Indeed, acute (1, 5 and 50 mg/kg) and chronic (1 mg/kg) CBD enhanced PPI in male C57BL/6JArc mice without concomitant alteration in startle reactivity [14], while acute (1–15 mg/kg) CBD had no effect on PPI in male Swiss mice but increased the startle response [15]. This suggests a dose- and strain-dependent effect of CBD on ASR. However, additional research has to investigate this phenomenon further, as a recent study in rats suggests ASR-suppressing properties for CBD [40]. Interestingly, PPI was decreased in vehicle-treated *Nrg1* TM HET mice only when tested after cessation of CBD treatment, reflecting the elusive and protocol-dependent nature of a definitive baseline PPI phenotype in *Nrg1* TM HET mice [26], [41], [42].

### Pharmacokinetics of CBD

Accumulation of CBD in blood in a dose-dependent manner was reflected by comparable CBD levels after treatment was withheld for 48 h in both *Nrg1* TM HET and WT mice. Recent data suggest that a single dose of 120 mg/kg CBD administered i.p. to mice is undetectable in brain and plasma after 24 h using tandem liquid chromatography mass spectrometry [43]. However, our method detected CBD in whole blood 48 h after the last i.p. injection suggesting that CBD accrues in the body with repeated exposure. This effect of repeated administration might be due to CBD's hydrophobicity and would be similar to the characteristics of THC that is retained in lipid rich tissues [44], [45]. It is possible that residual CBD affected the behavioural performance of test mice during the WH day. However, the lack of difference between residual CBD levels suggests that the behavioural and receptor binding differences between genotypes are not due to simple differences in CBD blood concentration.

### CBD alters 5-HT_2A_ and GABA_A_ receptor binding in a genotype-specific manner

*Nrg1* TM HET mice displayed a baseline decrease in 5-HT_2A_ receptor binding in the substantia nigra. CBD (50 mg/kg) selectively reduced binding of 5-HT_2A_ receptors in the substantia nigra in WT mice and increased binding of GABA_A_ receptors in the retrosplenial granular cortex in mutant mice. These changes in 5-HT_2A_ and GABA_A_ occur in areas relevant to both the behavioural changes we have observed and to the pathophysiology of schizophrenia. Midbrain 5-HT_2_ receptors regulate striatal dopaminergic transmission [46], [47]. Therefore, reduced 5-HT_2A_ receptor density might be related to the hyperactivity that occurs in *Nrg1* TM HET mice. On the other hand, increased 5-HT_2A_ binding in the cortex of adult *Nrg1* TM HET mice [48] suggests that changes in these receptors in response to *Nrg1* mutation may occur in a region- and thus functionally specific manner. The GABA_A_ binding increase in the granular retrosplenial cortex of mutant mice treated with CBD (50 mg/kg) occurred in the absence of baseline binding differences. Since the retrosplenial cortex mediates emotional responsivity and processing of emotional salience [49], [50], it is tempting to speculate that the selective increase in social interaction by the same dose of CBD in mutant mice is related to the change in GABA_A_ binding. Indeed, GABA_A_ agonists exert anxiolytic-like effects in the social interaction test [51].

Brains were collected from mice that had not received CBD for 48 h. It is possible that the binding changes we observed are related to a withdrawal-like state induced by withholding CBD treatment for 48 h, rather than to changes induced by the long-term treatment itself. However, the presence of CBD in blood at the same time as the collection of brain tissue and the absence of any withdrawal symptoms in the test cohorts suggest that withdrawal effects are unlikely.

It is tempting to speculate on mechanisms underlying the unmasking of certain effects of CBD, such as anxiolytic-like effects in *Nrg1* mutant mice, since our data would suggest that the normal functioning of Nrg1 might suppress some effects of CBD. For example, *Nrg1* mutation might enhance the responsiveness of targets of CBD suggested to be involved in modulating anxiety, such as the 5-HT_1A_ receptor [12], [52]. We did not observe increased 5-HT_1A_ receptor binding in our mutants; nevertheless this does not rule out enhanced signal transduction from this receptor that might be related to altered Nrg1 function. CBD might also modify behaviour via altering endocannabinoid tone, e.g. via inhibition of the anandamide hydrolysis enzyme fatty acid amide hydrolase [24]. Given that inhibition of this enzyme has documented anxiolytic effects [53], it would be worth investigating common signalling pathways between Nrg1 and the endocannabinoid system.

In conclusion, we present the novel findings that CBD alters specific aspects of the behavioural phenotype and brain receptor binding density in *Nrg1* TM HET mice. CBD did not reverse several of the schizophrenia-related behavioural features of mutant mice, namely hyperactivity, reduced PPI and reduced 5-HT_2A_ receptor density, although unlike those treated with vehicle, mutants treated with higher doses of CBD failed to express significant hyperactivity. CBD selectively enhanced social behaviour, prepulse inhibition, and retrosplenial GABA_A_ binding in *Nrg1* TM HET mice, supporting its potential therapeutic value in treating specific symptoms of schizophrenia. It appears that mutation in *Nrg1* unmasks this behavioural effect of CBD, whereas intact *Nrg1* is crucial for its anxiolytic effects. Future research has to investigate the effectiveness of long-term treatment with established antipsychotic drugs in this animal model to enable the evaluation of the current findings for schizophrenia therapy.

## Materials and Methods

### Animals

Male heterozygous *Nrg1^+/−^* (*Nrg1* TM HET) and wild type-like control *Nrg1^+/+^* (WT) littermates [25] aged 21±3 weeks were used as males exhibit a stronger sensitivity to cannabinoids than females [29], [32]. Standard social interaction opponents were age-matched male A/JArc mice (Animal Resources Centre, Canning Vale, Australia). Mice were pair-housed with limited environmental enrichment [mouse igloo (Bioserv, Frenchtown, USA) and a metal ring in the cage lid] under a 12∶12 h light∶dark schedule. Food and water were available *ad libitum*. Research and animal care procedures were approved by the University of New South Wales Animal Care and Ethics Committee in accordance with the Australian Code of Practice for the Care and Use of Animals for Scientific Purposes (ACEC approval number: 08/28A).

### Drug treatment

CBD (THC Pharm GmbH, Frankfurt, Germany) was suspended in a 1∶1∶18 mixture of ethanol∶Tween 80®∶saline. Mice received 21 consecutive daily intraperitoneal (i.p.) injections of vehicle (1∶1∶18 ethanol∶Tween 80®∶saline mixture) or CBD (1, 50 or 100 mg/kg) at a volume of 10 ml/kg as published previously [14].

### Behavioural testing

Treatment injections commenced 30 min after the start of the light cycle. Mice were behaviourally tested 30–45 min post injection on the first day of treatment (“acute” group) and on intermittent days throughout repeated treatment (“long-term” group), and after two days after the final dose of CBD treatment [“treatment withheld (WH day)” group] (Table 4). On behavioural testing days injections were staggered within the light cycle to standardise intervals between injection and testing [i.e. as groups of test mice (maximum of four mice at a time) were run consecutively, injections were administered the requisite number of minutes prior to testing to ensure consistent intervals between treatment and testing]. Mice were returned to the home cage following injection and behavioural testing. Environmental odours were removed from test apparatus between trials with 70% ethanol. The test order was based on an earlier study [14].

**Table 4:** Test biography of mice.

Test/Treatment Day	Test
**1**	Light-dark test (LD), open field (OF), prepulse inhibition (PPI)
**13**	OF
**14**	Novel object recognition test (NORT) habituation trials 1–2
**15**	LD, NORT habituation trial 3
**16**	NORT habituation trials 4–5
**17**	NORT test trials 1–2
**19**	Social interaction
**21**	PPI
**WH**	OF, LD, PPI

126 mice were injected with either vehicle or CBD (1, 50 or 100 mg/kg body weight) once daily from test day 1–21 (n = 14–17). Animals were tested again in OF, LD and PPI 48 h after the completion of the chronic CBD administration (WH day = test day 23).

#### Light–dark test (LD)

Mice were placed into the opening of a dark box insert in an open field (OF) activity chamber (41×41 cm; Tru-Scan Photo Beam Activity System: Coulbourn Instruments, Whitehall, USA) for 10 min. Horizontal activity (distance travelled) for both light (∼70 lx) and dark chambers (<5 lx) was measured by the Tru-Scan system and ANY-maze™ video tracking software (Stoelting Co., Wood Dale, USA; light chamber only). Time in the dark chamber was interpolated by subtraction of time in the light chamber (measured by ANY-maze) from the total test time. The ratio of distance travelled in the light compartment to total distance travelled (distance ratio) and time spent in the light compartment were taken as measures of anxiety.

#### Spontaneous locomotor activity

Locomotor activity was measured in the OF chamber for 10 min. Distance travelled and vertical activity (*rearing*) in central and peripheral zones (centre coordinates 7.6 cm×7.6 cm from the periphery) were measured by Tru-Scan and ANY-maze™ software. The ratio of central to total distance travelled (distance ratio) and time spent in the centre were taken as measures of anxiety [54].

#### Novel object recognition test (NORT)

The distinction between familiar and unfamiliar objects is an index of recognition memory [55], [56]. Mice were habituated to the empty NORT apparatus (grey perspex arena; 35×35×30 cm) for 5 min twice daily for 2 days. The following day, mice were habituated twice to the test procedure (i.e. exposure to identical objects placed in opposite corners). The next day, mice were placed in the arena for 10 min, which contained two novel identical objects, and allowed to explore freely (test trial 1). In test trial 2, 60 min later, the arena contained one copy of these objects (familiar object) and one novel object in the same positions as in test trial 1. Object exploration was scored for 5 min by the behaviours *nosing* (when the mouse directed its nose to an object at a distance of ≤1 cm) and *rearing* on the object. Data from NORT are not reported since the performance of WT mice at the novel object was not significantly different from chance (i.e. no indication of successful learning of objects).

#### Social interaction (SI)

SI between rodent pairs is used to measure anxiety-like behaviours [57]. Furthermore, reduction in SI models aspects of social withdrawal, which is also observed in schizophrenia patients [58]. Test mice and untreated, weight-matched (i.e. A/J body weight<test mice body weight) standard opponents were placed in opposite corners of the arena, which was used for NORT testing. Frequency and duration of the active socio-positive behaviours *nosing* [i.e. test mouse sniffs at the opponent's body, which is in close proximity to the test mouse (<1 cm)], *anogenital sniffing*, *allogrooming*, *following* and *climbing over/under* were scored for 10 min. Distance travelled was measured by ANY-maze™.

#### Prepulse inhibition (PPI)

PPI, an operational measure of sensorimotor gating impaired in schizophrenia patients [59], is the attenuation of the startle response by a non-startling stimulus (prepulse) presented before the startling stimulus (pulse). Startle reactivity was measured for 200 ms post pulse onset using SR-LAB startle chambers (San Diego Instruments, San Diego, USA). The PPI test consisted of 5 min acclimatisation to 70 dB background noise, followed by 105 trials in a pseudorandom order as published previously [41]: 5×70 dB trials (background); 5×80 dB trials; 5×100 dB trials; 15×120 dB trials (startle) and 5 sets of 15 trials comprising a prepulse of either 74, 82 or 86 dB presented 32, 64, 128, 256 or 512 ms (variable interstimulus interval; ISI) prior to a startling pulse of 120 dB (PPI response). The intertrial interval varied randomly from 10–20 s. Acoustic startle response (ASR) was calculated as the mean amplitude to the middle 5 startle trials to eliminate habituation effects [60]. Percentage PPI (% PPI) was calculated as [(mean startle response – PPI response)/mean startle response]×100. % PPI was averaged across ISIs.

### Detection and quantification of CBD in whole blood samples

Trunk blood was collected in EDTA-coated tubes immediately after PPI testing on WH day. CBD concentration was measured as described previously [44] with modifications for CBD analysis [61], [62]. 50 µl of D3-CBD (0.25 mg/L; PM Separations, Capalaba, Australia) internal standard solution was added to 0.5 ml trunk blood. Acetate buffer was added (pH 4.0) and CBD extracted with 1-chloro-butane solvent. Following complete drying under nitrogen, samples underwent derivatisation of the polar functional groups (COOH, OH) with bis(trimethylsilyl)trifluroacetamide. Quantification (1.25 ng/ml limit of quantification) of the derivatised extract was performed by gas chromatography-mass spectrometry (GC-MS) (Shimadzu 2010 Plus system: Shimadzu Scientific Instruments, Rydalmere, Australia).

### Receptor autoradiography

Brains from a subset of sacrificed mice (n = 4–5 per factor; selected randomly) were dissected 48 h after the last CBD injection, snap frozen and stored at −80°C. Coronal sections (14 µm) were cut and thaw-mounted onto slides. Specific receptors were chosen to investigate the effects of CBD treatment on the endocannabinoid system (i.e. CB_1_) and to determine the impact of CBD on a selection of central neurotransmitter systems relevant to schizophrenia (i.e. NMDA, 5-HT_1A_, 5-HT_2A_ and GABA_A_). For analysis, brain regions with relevance to schizophrenia and the endocannabinoid system were chosen in particular.

#### Autoradiographic binding

For CB_1_ receptors, sections were incubated for in 50 mM Tris-HCl buffer (pH 7.4) containing 5% bovine serum albumin (30 min) then in the same buffer containing 10 nM [^3^H]CP-55,940 (168 Ci/mmol; Perkin Elmer, Boston, USA) in the presence (non-specific binding) or absence (total binding) of 10 µM CP 55,940 [63].

For 5-HT_1A_ receptors, sections were incubated in 50 nM Tris-HCl buffer (30 min) then in the same buffer containing 5 nM [^3^H] WAY-100635 (83 Ci/mmol; Perkin Elmer) and 10 M pargyline in the presence (non-specific binding) or absence (total binding) of 10 µM 5-HT (150 min) [64].

For 5-HT_2A_ receptors, sections were incubated in 170 mM Tris-HCl buffer (15 min) then in the same buffer containing 4 nM [^3^H]ketanserin (88 Ci/mmol; Perkin Elmer) in the presence (non-specific binding) or absence (total binding) of 2 µM spiperone (120 min) [65].

For NMDA receptors, sections were incubated in 30 mM HEPES buffer (pH 7.5) containing 100 µM glycine, 100 µM glutamate, 1 mM EDTA and 20 nM [^3^H]MK-801 (17.1 Ci/mmol; Perkin Elmer) in the presence (non-specific binding) or absence (total binding) of 20 µM MK-801 (2.5 h) [66].

For GABA_A_ receptors, sections were incubated in 50 mM Tris-HCl buffer (pH 7.0) (3×5 min) then in the same buffer containing 3 nM [^3^H]muscimol (29.5 Ci/mmol) in the presence (non-specific binding) or absence (total binding) of 100 µM GABA (40 min) [67].

All sections were washed in ice-cold buffer, dipped in distilled water and air dried.

#### Quantification

Slides were exposed to Kodak BioMax MR film. Developed films were analysed using a computer-assisted image analysis system, Multi-Analyst, connected to a GS-690 Imaging Densitometer (Bio-Rad, Hercules, USA). Binding quantification was performed by measuring the average density in brain regions identified using a mouse brain atlas [68] in 2–3 adjacent sections and comparing the values against autoradiographic standards (Amersham: GE Healthcare, Buckinghamshire, UK).

### Statistical analysis

Behavioural measures and binding density were analysed with two-way analysis of variance (ANOVA) (between-subjects factors: ‘treatment’ and ‘genotype’) to distinguish between acute (day 1), long-term (days 13–21), and treatment withheld (day 23) effects. Repeated measures (RM) three-way ANOVAs were used for NORT [within-subjects factor: ‘object’ (novel or familiar)] and PPI (within-subjects factor: ‘prepulse intensity’). Initial ANOVAs were followed by two- or one-way ANOVAs split by the corresponding factor(s) if appropriate as published previously [29]–[32]. Differences between CBD doses were determined with Dunnett's post-hoc test whereas body weight was compared using an unpaired t-test. Data are presented as means ± standard error of the mean (S.E.M.). Main effects were regarded as statistically significant when *P*<0.05. A total of 126 mice were tested (n = 14–17). Degrees of freedom, F-values and *P*-values are shown for three- and two-way ANOVAs (* *versus* WT receiving corresponding treatment; # *versus* vehicle of corresponding genotype) are presented. In case malfunction of software or test equipment occurred, data were excluded, resulting in altered degrees of freedom for some analyses. Analysis was performed using SPSS 17.0.
